# Serum Vitamin D Insufficiency in Hospitalized Full-Term Neonates at a Tertiary Hospital in Eastern China

**DOI:** 10.3389/fped.2022.878992

**Published:** 2022-05-26

**Authors:** Huawei Wang, Yiming Du, Zhixin Wu, Haifeng Geng, Xueping Zhu, Xiaoli Zhu

**Affiliations:** ^1^Department of Neonatology, Children's Hospital of Soochow University, Suzhou, China; ^2^Department of Intervention, The First Affiliated Hospital of Soochow University, Suzhou, China

**Keywords:** Vitamin D, prevalence, insufficiency, deficiency, neonate

## Abstract

**Objective:**

This study explored the status of serum vitamin D in hospitalized full-term neonates at a tertiary hospital in eastern China.

**Methods:**

A prospective study was conducted among 471 hospitalized full-term neonates at the Children's Hospital of Soochow University between January 1 and June 20, 2020. Perinatal clinical data, serum 25-hydroxyvitamin D (25(OH)D_3_), laboratory examinations, serum calcium levels, and immune function were obtained and analyzed. We explored and analyzed the risk factors for vitamin D insufficiency or deficiency, and we also attempted to determine correlations between vitamin D and its influence on immunity.

**Results:**

The mean serum 25(OH)D_3_ was 33.65±6.07ng/ml.The prevalence of vitamin D insufficiency was 28.24%,vitamin D sufficiency was 71.76%, there was no vitamin D deficiency. The serum 25-(OH)D_3_ in singleton neonate was higher than twins or multiple infants (*t* = −10.918, *P* = 0.000). The serum 25-(OH)D_3_ were higher in neonates who born in spring and summer than in winter (H = 13.443, *P* = 0.001). The serum 25-(OH)D_3_ in appropriate for gestational age (AGA) and large for gestational age (LGA) neonates were higher than small for gestational age (SGA) (H = 7.686, *P* = 0.021). The serum 25-(OH)D_3_ were higher in neonates whose mothers had no underlying diseases than those with certain immunological and infectious diseases (F = 12.417, *P* = 0.000). The serum 25-(OH)D_3_ in neonates whose mothers had none or one perinatal complication were higher than those with two or more (F = 13.299, *P* = 0.000). The neonates with eosinophils ≤5% or normal platelet counts or serum Ca^++^ ≥0.9 mmol/L have higher serum 25-(OH)D_3_. Neonates born in winter were at risk for vitamin D insufficiency, and the incidence of infectious pneumonia, sepsis, cytomegalovirus infection, and hypocalcemia in the vitamin D insufficiency group were higher than sufficiency group (*P* < 0.05). The serum CD3+, CD3+CD4+, and IgA levels in vitamin D sufficiency neonates were significantly higher than those in insufficiency group (*P* < 0.05).

**Conclusion:**

The prevalence of vitamin D insufficiency was 28.24%, and birth in winter was a risk factor for vitamin D insufficiency in hospitalized full-term neonates in Suzhou area. Neonates with infectious pneumonia, sepsis, cytomegalovirus infection, and hypocalcemia exhibited a high risk of vitamin D insufficiency. The serum CD3^+^, CD3^+^CD4^+^, and IgA levels in neonates with vitamin D insufficiency were lower.

## Introduction

Vitamin D is key hormone that regulates many essential physiological functions. Vitamin D insufficiency or deficiency is a global health problem which is associated with a variety of diseases, including infectious diseases, metabolic syndrome, and critical illnesses (1). Vitamin D insufficiency or deficiency is very common in populations around the world. It has been reported that most elderly men and women in high-income countries including U.S, Canadian, and European still living in the community are vitamin D deficient (2, 3). Vitamin D deficiency is also common in low-income countries such as Middle East, India, Africa, and South America (4, 5). Children, young and middle-aged adults are at equally high risk for vitamin D deficiency and insufficiency worldwide. The incidence rates and severity of vitamin D insufficiency and deficiency are higher in infants (6–8). A reduction in vitamin D intake and absorption, decreased synthesis, and increased activated 1,25- dihydroxyvitamin D_3_ (1,25(OH)2D_3_) can lead to severely low levels of vitamin D.

Risk factors for vitamin D deficiency in infant include breast-feeding without vitamin D supplementation and maternal vitamin D deficiency. The infant can remain vitamin D sufficient for several weeks after birth, as long as the mother was vitamin D sufficient. However, most pregnant women are vitamin D deficient or insufficient (9, 10). Lee et al. reported that 76% of mothers and 81% of newborns had a vitamin D deficiency at birth, despite the mothers ingested about 600 IU/d of vitamin D and consumption of two glasses of milk during the period of pregnancy (11).

The vitamin D insufficiency or deficiency is often associated with severe infectious diseases, multiple organ failure, and even mortality (12, 13). Vitamin D signaling imposed a key regulator of immunity in human beings. Vitamin D, also could induce expression of antibacterial proteins which is able to support increased bacterial killing in a variety of cell types (14, 15).

We conducted this study in the Suzhou area of East China to investigate the prevalence of vitamin D insufficiency in hospitalized full-term neonates and to evaluate the factors associated with vitamin D insufficiency. and to explore the relation of vitamin D insufficiency with immunocompromise.

## Methods

### Study Design

This study included infants admitted to the neonatal department of the Children's Hospital of Soochow University from January 1 to June 20, 2020. The inclusion criteria were (1) admission age ≤28 days, (2) gestational age ≥37 weeks and <42 weeks, (3) vitamin D and calcium were not supplemented before hospitalization. Exclusion criteria were (1) infants were treated at other hospitals or re-hospitalized, (2) meconium aspiration syndrome (MAS), (3) neonatal acute respiratory distress syndrome (NRDS), (4) complex congenital heart disease and congenital malformation, (5) maternal autoimmune diseases, and (6) discharge against medical advice.

This study was approved by the Ethics Committee of the Children's Hospital of Soochow University (approval number: 2020061). The parents of all infants provided written informed consent.

### Diagnostic Criteria and Grouping

Evaluation criteria for vitamin D were adopted according to the Endocrine Society Clinical Practice Guidelines for 25-(OH)D_3_ levels: deficiency (<20 ng/mL), insufficiency (20–30 ng/mL), and sufficiency (≥30 ng/mL) (16). We divided the neonates into vitamin D insufficiency and sufficiency groups based on their serum 25-(OH)D_3_ levels. Suzhou experiences four seasons: winter (December–February), spring (March–May), summer (June–August), and autumn (September– November).

### Clinical Variables

The following data and clinical indices were retrieved from the hospital records: maternal conditions, gestational age, sex, birth weight, age at admission, mode of delivery, perinatal complications (including gestational diabetes, gestational hypertension, gestational anemia, preeclampsia, amniotic fluid contamination, and prenatal infection), and other maternal diseases; laboratory examinations at admission: white blood cell (WBC count [10^9^/L]), hemoglobin (Hb [g/L]), eosinophil ratio (%), platelet count (×10^9^/L), C-reactive protein (mg/L), procalcitonin (ng/mL), and serum calcium (mmol/L).

### Analytical Biomarker Determination

Blood samples were collected on the day of admission and stored at −80°C. Serum 25(OH)D_3_ levels were measured using the Vit k Immune Diagnostic Assay System (Pomade Technology Co. Ltd, Beijing, China).

### Statistical Analysis

SPSS 25.0 was used for statistical analyses. Categorical variables were analyzed using either the chi-square test or Fisher's exact test. Normally distributed variables were represented as means ± standard deviations and analyzed using the independent *t*-test. Non-normally distributed variables were represented as medians ± interquartile ranges [M (P25, P75)], and analyzed using non-parametric tests. Significant factors were identified and multivariate logistic regression analysis was performed to evaluate the risk factors for vitamin D insufficiency. Statistical significance was set at *p* < 0.05.

## Results

### Patient Enrollment and Characteristics

A total of 471 full-term neonates (male: 290 [59.44%], female: 191 [40.56%], mean gestational age: 39.23 ± 1.06 weeks, median admission age: 5.00 [range: 2.37–13.00] days, mean birth weight: 3389.13 ± 411.16 g, mean admission weight: 3400.78 ± 515.50 g, singleton: 446 [94.69%], twins: 23 [5.31%]) admitted to the Department of Neonatology, Children's Hospital of Soochow University from January 1, 2020 to June 30, 2020, who underwent 25-(OH)D_3_ tests, were enrolled in this study.

### Analysis of Vitamin D Levels and the Influencing Factors

The mean 25-(OH)D_3_ level of hospitalized full-term neonates was 33.65 ± 6.07 ng/mL. The prevalence of vitamin D insufficiency was 28.24% (133 cases), and that of vitamin D sufficiency was 71.76% (338 cases); there was no vitamin D deficiency.

The serum 25-(OH)D_3_ level in singleton neonates was significantly higher than that in twins or multiple infants (*t* = −10.918, *P* = 0.000). The serum 25-(OH)D3 levels of the neonates born in spring and summer were significantly higher than those born in winter (H = 13.443, *P* = 0.001). The serum 25-(OH)D_3_ in appropriate for gestational age and large for gestational age neonates were significantly higher than that in small for gestational age (H = 7.686, *P* = 0.021). There were no statistically significant differences in serum 25-(OH)D_3_ levels between the sexes, delivery modes, conception modes, admission age, and postnatal feeding modes among the two groups of neonates (*P* > 0.05) (Table 1).

**Table 1:** Patient characteristics and clinical outcomes.

**Characteristic**	**Number of cases [n (%)]**	**25(OH)D_**3**_(ng/mL)**	**t/H/F**	***P*-value**
Sex				
Male	280(59.44)	33.41 ± 5.73	0.997^a^	0.307
Female	191(40.56)	33.99 ± 6.55		
Delivery mode				
Vaginal delivery	301(63.91)	34.02 ± 6.40	1.782^a^	0.075
Cesarean	170(36.09)	32.98 ± 5.40		
Number of births				
Singleton	446 (94.69)	34.00 ± 6.02	10.918^a^	0.000
Twins or multiple births	23 (5.31)	27.42 ± 2.65		
*In vitro* fertilization (IVF)			
Yes	12 (2.55)	31.63 ± 4.47	1.166^a^	0.244
No	459 (97.45)	33.70 ± 4.47		
Time of admission (days)			
≤ 7days	290 (61.57)	33.78 ± 6.29	0.590^a^	0.555
>7days	181(38.43)	33.44 ± 5.71		
Birth season				
Winter^*^	157 (33.33)	32.29 ± 4.52	13.443^b^	0.001
Spring	228 (48.41)	34.78 ± 6.76		
Summer	86 (18.26)	33.12 ± 6.12		
Birth weight				
SGA^*^	15 (3.18)	29.85 ± 3.45	7.686^b^	0.021
AGA	434 (92.14)	33.83 ± 6.17		
LGA	22 (4.67)	32.73 ± 4.44		
Feeding type				
Un-feeding	23 (4.88)	33.66 ± 5.69	0.420^c^	0.739
Breastfeeding	224 (47.56)	33.92 ± 6.08		
Formula	62 (13.17)	33.07 ± 5.05		
Mixed Feeding	162 (34.39)	33.48 ± 6.49		

*Data are presented as number, number (%), mean (standard deviation)*.

*a, the t-test; b, the Kruskal–Wallis H test; and c, the variance analysis*.

* *indicates statistically significant differences between the groups*.

*SGA, small for gestational age; AGA, appropriate for gestational age; LGA, large for gestational age*.

The serum 25-(OH)D_3_ levels in neonates whose mothers had no underlying diseases were significantly higher than those born to mothers with immunological diseases (including rheumatoid arthritis, systemic lupus erythematosus, hypothyroidism, and rheumatic heart disease) and infectious diseases (including syphilis, hepatitis B, and prenatal fever) (F = 12.417, *P* = 0.000). Serum 25-(OH)D_3_ levels were significantly higher in neonates whose mothers had infectious diseases than in those born to mothers with immunological diseases (*t* = −3.136, *P* = 0.004). The serum 25-(OH)D_3_ level in neonates whose mothers had none or one perinatal complication were significantly higher than those with mothers having two or more complications (F = 13.299, *P* = 0.000). There was no statistically significant difference in maternal age (*P* > 0.05) between the groups (Table 2).

**Table 2:** Maternal characteristics.

**Characteristic**	**Number of cases [*n* (%)]**	**25(OH)D (ng/mL)**	**t/F**	***P*-value**
Mother's age, years				
≥35	55 (11.67)	33.58 ± 6.08	−0.608^a^	0.543
<35	416 (88.33)	34.12 ± 6.01		
Underlying disease^•^				
NO	443 (94.05)	33.98 ± 6.09	12.417^c^	0.000
Immune disease	10 (2.12)	26.80 ± 2.73		
Infectious disease	18 (3.83)	29.30 ± 1.48		
Gestational disease during pregnancy			
NO	327 (69.42)	33.71 ± 5.72	13.299^c^	0.000
1 complication	130 (27.60)	34.33 ± 6.60		
≥2 complications ^*^	14 (2.98)	25.76 ± 2.66		

*Data are presented as number, number (%), mean (standard deviation)*.

*a, the t-test; c, the variance analysis*.

• *The comparison between the two groups was statistically significant*.

* *indicates statistically significant differences between the groups*.

The serum 25-(OH)D_3_ level in neonates with eosinophil ≤5% was significantly higher than that in neonates with eosinophils >5% (T = 8.556, *P* = 0.000). Serum 25-(OH)D3 levels in neonates with normal platelet counts were significantly higher than in those with abnormal platelet counts (*t* = −2.412, *P* = 0.016). The serum 25-(OH)D_3_ in neonates with serum Ca^++^ ≥0.9 mmol/L was significantly higher than that in neonates with serum Ca^++^ <0.9 mmol/L (*t* = −7.589, *P* = 0.000). There were no statistically significant differences (*P* > 0.05) in vitamin D levels regarding WBC count, hemoglobin, C-reactive protein and procalcitonin at admission (Table 3).

**Table 3:** Results of laboratory examinations of neonates.

**Characteristic**	**Cases [*n*, (%)]**	**25(OH)D_**3**_(ng/mL)**	***t***	***P*-value**
White blood cell count (×10^9^ /L)				
>20 ×10^9^/L or <4 ×10^9^/L	183 (38.85)	33.32 ± 6.20	−0.907	0.365
Normal range	286 (61.15)	33.84 ± 6.01		
Hemoglobin (g/L)				
≥145 g/L	348 (73.89)	33.52 ± 6.24	0.649	0.439
<145 g/L	123 (26.11)	34.01 ± 5.56		
Eosinophil ratio (%)				
>5%	113(23.99)	29.72 ± 3.28	8.556	0.000
≤ 5%	358(76.01)	34.87 ± 6.12		
Platelets (×10^9^/L)				
>300 ×10^9^/L or <100 ×10^9^/L	225(47.77)	32.95 ± 5.84	−2.412	0.016
≤ 300 ×10^9^/L	246(52.23)	34.29 ± 6.23		
C-reactive protein (mg/L)				
≥8 mg/mL	423(89.81)	33.64 ± 6.15	0.061	0.951
<8 mg/mL	48(10.19)	33.70 ± 5.38		
Procalcitonin (ng/mL)				
≥0.5 ng/mL	107(22.71)	34.27 ± 6.27	1.217	0.224
<0.5 ng/mL	364(77.29)	33.46 ± 6.01		
Serum calcium ion (mmol/L)				
≥0.9 mmol/L	405(85.99)	34.23 ± 6.18	−7.589	0.000
<0.9 mmol/L	66(14.01)	30.04 ± 3.73		

*Data are presented as number, number (%), mean (standard deviation)*.

### Multifactorial Analysis of Vitamin D Insufficiency

Multivariate logistic regression analysis based on the statistically significant factors associated with vitamin D insufficiency, revealed that being born in winter was a risk factor for vitamin D insufficiency in full-term neonates (Table 4).

**Table 4:** Multivariate logistic regression analysis predicting the risk vitamin D insufficiency in full-term neonates.

**Variable**	**β**	**SE**	**Wald**	***P***	**OR**	**95% CI**
Birth in winter	1.138	0.258	19.458	40.000	0.320	0.193–0.531

### Comparison of Diseases Between Vitamin D Groups

The incidence rates of infectious pneumonia, sepsis, cytomegalovirus infection, and hypocalcemia were higher in the vitamin D insufficiency group than those in the sufficiency group (*P* < 0.05).

The incidence rates of enteritis, urinary tract infection, bacterial meningitis, pathological jaundice, hemolytic disease, vomiting, neonatal asphyxia, hydronephrosis, atrial septal defect, ventricular septal defect, and patent ductus arteriosus were not significantly different between the two groups (*P* > 0.05) (Table 5).

**Table 5:** Comparison of vitamin D sufficient and insufficient groups.

**Characteristics**	**Vitamin D insufficiency *n* (%)**	**Vitamin D sufficiency *n* (%)**	**χ^2^**	***P*-value**
Infectious diseases				
Infectious pneumonia	44 (33.08)	76 (22.48)	5.646	0.017
Enteritis	12 (9.02)	28 (8.28)	0.067	0.796
Urinary infection	18 (13.53)	35 (10.35)	0.966	0.326
Sepsis	7 (5.26)	5 (1.47)	4.862	0.044
Bacterial meningitis	2 (1.52)	5 (1.50)	0.000	0.984
CMV infection	7 (5.26)	6 (1.77)	4.393	0.036
Non-infectious diseases				
Pathological jaundice	40 (30.07)	80 (23.67)	2.063	0.151
Hypocalcemia	28 (21.05)	38 (11.24)	7.623	0.006
Hemolytic disease	9 (6.76)	32 (9.46)	0.876	0.349
Neonatal vomiting	6 (4.51)	17 (5.02)	0.055	0.814
Neonatal asphyxia	3 (2.25)	7 (2.07)	0.016	0.900
Hydronephrosis	5 (3.75)	13 (3.84)	0.002	0.965
ASD	35 (26.31)	90 (26.62)	0.005	0.945
VSD	6 (4.51)	14 (4.14)	0.032	0.858
PDA	3 (2.25)	12 (3.55)	0.184	0.471
Subarachnoid hemorrhage	20 (15.03)	47 (13.91)	0.099	0.770

*Data are presented as number, number (%)*.

*CMV, cytomegalovirus; ASD, atrial septal defect; VSD, ventricular septal defect; PDA, patent ductus arteriosus*.

### Correlation of Serum Vitamin D With Immunological Indicators

Among the 471 full-term neonates, 380 exhibited immunological indicators. The serum CD3+, CD3+CD4+, and IgA levels in the vitamin D sufficient group were significantly higher than those in the insufficiency group (*P* < 0.05). However, there were no significant differences in serum CD3+CD8+, CD4+/CD8+, CD3-CD19+, CD3-CD16+CD56+, complement C3, complement C4, IgM, and IgG between the two groups (*P* > 0.05) (Table 6).

**Table 6:** Correlation of serum vitamin D with immunological indicators.

**Characteristics**	**Vitamin D insufficiency (*n* = 96)**	**Vitamin D sufficiency (*n* = 284)**	**t/U**	***P*-value**
CD3^+^ [%, ($\bar{x}\pm$ s)]	78.97 ± 6.70	82.75 ± 6.99	−3.646^a^	0.000
CD3^+^CD4^+^ [%, ($\bar{x}$ ± s)]	57.61 ± 6.75	59.90 ± 7.52	−2.242^a^	0.026
CD3^+^CD8^+^ [%, ($\bar{x}$ ± s)]	21.14 ± 5.27	22.10 ± 6.23	−1.353^a^	0.177
CD4^+^/CD8^+^ [%, ($\bar{x}$ ± s)]	2.93 ± 1.03	2.99 ± 1.21	−0.474^a^	0.636
CD3^−^CD19^+^ [%, ($\bar{x}$± s)]	9.46 ± 5.09	8.99 ± 4.42	−1.288^a^	0.199
CD3^−^CD16^+^CD56^+^ [%, ($\bar{x}$ ± s)]	9.56 ± 5.57	8.97 ± 5.75	−1.70^a^	0.142
complement C3 [g/L, ($\bar{x}$ ± s)]	0.63 ± 0.15	0.66 ± 0.16	−1.466^a^	0.144
complement C4 [g/L, ($\bar{x}$ ± s)]	0.15 ± 0.05	0.15 ± 0.06	−0.876^a^	0.381
IgA [g/L, ($\bar{x}$ ± s)]	0.04 ± 0.02	0.05 ± 0.03	−3.608^a^	0.000
IgM [g/L, M(P_25_, P_75_)]	0.15(0.09, 0.21)	0.13(0.07, 0.23)	−0.218^d^	0.828
IgG [g/L, ($\bar{x}$ ± s)]	8.49 ± 2.02	8.47 ± 1.87	−0.511^a^	0.586

*Data are presented as mean (standard deviation), or median (interquartile range)*.

*a, t-test; b, Kruskal–Wallis H-test*.

## Discussion

This study indicates that vitamin D insufficiency is associated with being born in winter. Moreover, we observed that the prevalence of vitamin D insufficiency in neonates with infectious pneumonia, sepsis, cytomegalovirus infection, and hypocalcemia was higher and the serum CD3+, CD3+, CD4+, and IgA levels were lower in neonates with vitamin D insufficiency.

In our prospective study, we found that the prevalence of vitamin D insufficiency was 28.24%, the vitamin D sufficiency was 71.76%, and there was no vitamin D deficiency in our hospital. A similar study in Boston, northeastern USA, revealed that the prevalence of serum vitamin D insufficiency (25-(OH)D3, 20–30ng/mL) in neonates with gestational age ≥37 weeks was 24.7% (17). The incidence of vitamin D insufficiency in preterm neonates was not included in this study. There are regional and lactation differences regarding neonatal serum vitamin D and associated with vitamin D deficiency during pregnancy (18). Suzhou city is located near the ocean, latitude 30°47′-32°2′ (mid-latitude region), with warm weather and adequate sunlight. The region has a flourishing economy and commerce. During pregnancy, women consciously ensure that they receive sufficient vitamin D by supplementation, which is why we found no vitamin D deficiency in hospitalized full-term neonates. Also we thought the pregnant mother in our region get enough Vitamin D supplement, further, we need to continue exploring the serum of maternal Vitamin D influence on the infants.

Our findings revealed that being born in winter was an independent risk factor for vitamin D insufficiency in full-term neonates. We all know that the major source of vitamin D for children and adults is exposure to natural sunlight (19). This widely accepted association between vitamin D insufficiency status and the season of birth was reported by Mosayebi et al. (20). They found that the majority of neonates with vitamin D insufficiency were born on cold days during the winter, and the vitamin D status was closely related to the season of birth. The correlation between vitamin D status and the season of birth has also been reported by Khuri-Bulos et al. (21) who showed that most neonates with low levels of vitamin D were born during the winter months. However, other studies did not identify a specific relationship between the season of birth and serum vitamin D levels in neonates (22). It appears that exposure to sunlight is not a critical factor for serum vitamin D status. The synthesis of vitamin D is affected by various factors such as latitude, season, air pollution, and clothing. Vitamin D is primarily synthesized by exposure of the skin to direct sunlight. Receiving breast milk, which chronically contains low levels of vitamin D and certain customs of ancient cultures that reduce direct sun exposure would contribute to the deficiency or insufficiency of vitamin D in neonates (23). Consequently, some research has shown that vitamin D was not being produced, not only in regions at latitudes of 45° or higher (such as North America and Europe), but also during special periods, including winter, which may last 6 months of the year or longer (24). Moreover, some studies revealed other factors that may affect the vitamin D status in neonates, such as mothers with restricted sun exposure inducing maternal vitamin D deficiency, preterm babies, and small for gestational age (25).

Low serum vitamin D levels in neonates are associated with rickets, bronchopulmonary dysplasia (BPD), respiratory tract infections, and other diseases (26–28). A previous study revealed that neonates with vitamin D deficiency or insufficiency are at greater risk of developing serious infection and sepsis, and lower serum vitamin D levels in pregnant mothers are closely linked with increased risk of sepsis in neonates (29). A study also showed that 25 hydroxy vitamin D level of children in the NRDS group was significantly lower than that of the non-NRDS group (SMD = −0.51, 95%CI: −0.63 to −0.39, *p* ≤ 0.05). Suggests that vitamin D deficiency is very likely to be a high-risk factor of NRDS, and reasonable vitamin D supplementation during pregnancy and after birth is of great significance (30). In other related fields, a study in Taipei revealed that the prevalence of vitamin D deficiency was 59%, and only 10% of critically ill patients had a vitamin D level >30 ng/mL. They also found that most critically ill patients had a median vitamin D level <20 ng/mL during their stay at the intensive care units (31).

In our study, the serum CD3^+^, CD3^+^CD4^+^, and IgA levels in neonates with vitamin D insufficiency were lower. Vitamin D has other functions as well, including modulating activated B and T lymphocytes, insulin, and thyroid-stimulating hormone secretion (32). Vitamin D potentially influences human immunity, including the induction of antimicrobial peptides (AMPs) and suppression of T-cell proliferation. Infants with rickets are at risk of developing diseases unrelated to calcium homeostasis, including immune disorders, and a study showed that immune production of 25-(OH)D is regulated by factors that are not linked to calcium homeostasis. Local synthesis of 25-(OH)D and its downstream signaling are integral components of innate immune responses to microbes (33). Another study reported that locally produced 25-(OH)D activated the transcription of certain signaling pathways and genes related to innate immunity. In laboratory studies, the gene encoding CD14, the co-receptor of the pattern recognition receptor toll-like receptor-4, is strongly induced by 25-(OH)D (34). Clinical and epidemiological studies have reported that the immune protection induced by vitamin D could protect the body from some viruses, including hepatitis viruses, human immunodeficiency virus, and viral respiratory pathogens (35).

In our analysis, several limitations were noted. We did not identify whether suboptimal maternal vitamin D status directly contributed to the neonates. Further, our study did not include data on prenatal vitamin D supplementation and maternal 25(OH)D levels and therefore, did not facilitate better risk prediction regarding suboptimal vitamin D status.

## Conclusions

Vitamin D is key hormone that plays an important role in human health. Vitamin D insufficiency or deficiency is a global health problem which is associated with a variety of diseases. Results from this study suggest the prevalence of vitamin D insufficiency in neonates is high, and birth in winter was a risk factor for vitamin D insufficiency in hospitalized full-term neonates. Neonates with infectious pneumonia, sepsis, cytomegalovirus infection, and hypocalcemia exhibited a high risk of vitamin D insufficiency. The serum CD3^+^, CD3^+^CD4^+^, and IgA levels in neonates with vitamin D insufficiency were lower. We believe that suppling and remain the neonates Vitamin D sufficiency will improve initiatives for optimizing neonate care.

## Data Availability Statement

The original contributions presented in the study are included in the article/supplementary material, further inquiries can be directed to the corresponding authors.

## Ethics Statement

The studies involving human participants were reviewed and approved by Ethics Committee of the Children's Hospital of Soochow University. Written informed consent to participate in this study was provided by the participants' legal guardian/next of kin.

## Author Contributions

HW and YD participated in the study design and manuscript writing. ZW and HG collected clinical data collection and conducted data analysis. XuZ and XiZ supervised the design and execution of the study, performed the final data analysis, and contributed to the writing of the manuscript. All authors read and approved the final manuscript.

## Funding

This study was financially supported by the National Natural Science Foundation of China (No. 81971423, 81771626), Jiangsu Key Talent Program of Maternal and Child Health (No. FRC201731), Jiangsu Provincial Social Development Key General Project (No. BE2020658), and Jiangsu Provincial Health and Family Planning Commission Medical Research Project (No. ZD2021013).

## Conflict of Interest

The authors declare that the research was conducted in the absence of any commercial or financial relationships that could be construed as a potential conflict of interest.

## Publisher's Note

All claims expressed in this article are solely those of the authors and do not necessarily represent those of their affiliated organizations, or those of the publisher, the editors and the reviewers. Any product that may be evaluated in this article, or claim that may be made by its manufacturer, is not guaranteed or endorsed by the publisher.
